# How is active transport associated with children's and adolescents' physical activity over time?

**DOI:** 10.1186/1479-5868-8-126

**Published:** 2011-11-14

**Authors:** Alison Carver, Anna F Timperio, Kylie D Hesketh, Nicola D Ridgers, Jo L Salmon, David A Crawford

**Affiliations:** 1 Centre for Physical Activity and Nutrition Research, School of Exercise and Nutrition Sciences, Deakin University, Victoria, Australia

**Keywords:** Tracking, longitudinal, youth

## Abstract

**Background:**

As few longitudinal studies have examined how active transport is associated with physical activity among children and adolescents over time, and how active transport tracks through childhood and adolescence, it is important to understand whether physically active children retain their activity patterns through adolescence. This study aimed to examine (a) tracking of active transport and of moderate-to-vigorous physical activity (MVPA) across childhood and adolescence in two age cohorts; and (b) associations between active transport and MVPA at three distinct time-points, over five years.

**Methods:**

This longitudinal study of two cohorts aged 5-6 years (n = 134) and 10-12 years (n = 201) at baseline (T1), in Melbourne, Australia, gathered follow-up data at three (T2) and five years (T3). Walking/cycling to local destinations was survey-reported; while MVPA was recorded using accelerometers and mean time spent daily in MVPA on week days and on weekends was computed. Tracking of these behaviours was examined over five years using General Estimating Equations. Linear regression analyses were performed to examine associations between active transport and MVPA at each time-point.

**Results:**

Active transport tracked moderately among children (boys, β_s _= 0.36; girls, β_s _= 0.51) but not among adolescents. Physical activity tracked moderately (β_s _value range: 0.33-0.55) for both cohorts. Active transport was not associated with children's MVPA at any time-point, but was associated with adolescent boys' MVPA on week days at T1 (B = 1.37 (95% CI: 0.15, 2.59)), at T2 (B = 1.27 (95% CI: 0.03, 2.51)) and at T3 (B = 0.74 (95% CI: 0.01, 1.47)), and with adolescent girls' MVPA on week days (B = 0.40 (95% CI: 0.04, 0.76)) and on weekends (B = 0.54 (95% CI: 0.16, 0.93)) at T3 only.

**Conclusion:**

Active transport was associated only with boys' MVPA during early adolescence and with boys' and girls' MVPA during late adolescence. While active transport should be encouraged among all school-aged children, it may provide an important source of habitual physical activity for adolescent girls, in particular, among whom low and declining physical activity levels have been reported world-wide.

## Introduction

The decline in children's active transport (e.g., walking and cycling) to school and other destinations over recent decades is of public health concern [1,2]. The benefits of active transport for the whole population are multi-faceted and include reductions in carbon emissions, less noise from traffic, reduced consumption of fossil fuels and greater social interaction, as well as opportunities for habitual physical activity [3]. For young people, regular physical activity during childhood and adolescence has well-documented health benefits including reduced risk of cardiovascular disease, type 2 diabetes and obesity [4,5]. In addition, active transport may promote independence, exploration of the natural and built environments and the development of social skills, particularly if children are unaccompanied by adults [1,6].

In 2001, Tudor-Locke, Ainsworth and Popkin [7] identified active transport on the journey to school as a potential source of habitual physical activity. They called for research to understand how active transport to school contributes to overall physical activity levels, as well as longitudinal studies examining tracking of active transport through adolescence [7]. Since then, interest in this area has burgeoned. However, most evidence on this topic is from cross-sectional studies. To date, there is a paucity of longitudinal studies that specifically examine active transport across the transition from childhood to adolescence, and of studies that examine tracking of active transport behaviours. Tracking refers to the degree of stability of an individual's relative rank within a group over time [8].

A systematic review [9] of studies published between 2003 and 2008 that reported active transport to school and youth physical activity levels found that in 11 out of 13 studies, schoolchildren who walked or cycled to school were more physically active overall than those who used motorized modes of transport. More recently, a further systematic review [10] was conducted of studies published between 1980 and 2009 that reported associations between active transport to school and the following measures of fitness: cardiorespiratory fitness, muscular fitness, body composition and flexibility. Active transport was shown to be consistently associated with lean body composition and cardiorespiratory fitness [10].

One of the strongest barriers and most consistent correlates of walking and cycling to school is distance between home and school [11,12]. Hillman [6] reports that in England many children now travel greater distances to school as a result of parents being offered greater choice of schools. In an Australian study, the average distance of the most direct route to school was 2.3 (SD = 3.1) km [13]. Given that many children may not live within an easy walking or cycling distance from school, it may also be important to consider children's active transport to other destinations within their neighborhoods. Little is known about associations between active transport to all neighborhood destinations (not just school) and overall physical activity among youth. Active transport may provide more attractive options for informal, social physical activity for some adolescents, in particular girls, who prefer less focus on competition and ability levels [14].

As few longitudinal studies have examined how active transport is associated with physical activity among children and adolescents over time, and how active transport tracks through childhood and adolescence, it is important to understand whether physically active children retain their activity patterns through adolescence. This may guide the choice of age-groups to be targeted by interventions to promote physical activity. From a public heath perspective it is important to motivate inactive children/adolescents to be active and to motivate those who are active to remain active [15]. The aims of this study were to examine: (a) tracking of active transport and of moderate-to-vigorous physical activity (MVPA) across childhood and adolescence in two age cohorts; and (b) associations between active transport and MVPA at three distinct time-points, over five years.

## Methods

### Sample

Data were gathered as part of a longitudinal study called "Children Living in Active Neighbourhoods". Two cohorts of schoolchildren aged 5-6 years and 10-12 years at baseline (2001, T1) were followed up three (T2) and five (T3) years later. Originally designed as a cross-sectional study, at baseline, 295 children aged 5-6 years and 919 children aged 10-12 years (27%, 44% response rates, respectively) were recruited from 19 government primary schools across Melbourne, Australia. Participation rates for the first follow-up (T2) among those whose parents had agreed to future contact were 76% (n = 191) of the younger cohort, then aged 8-9 years, and 64% (n = 416) of the older cohort, then adolescents aged 13-15 years. Participation rates for the second follow-up (T3) among those who agreed to be re-contacted were 93% for children (n = 177) aged 10-11 years and 68% for adolescents (n = 326) aged 15-17 years, representing 60% and 35% of their respective cohorts at baseline. Ethics approval for this study was obtained from the Deakin University Ethics Committee, the Department of Education and Training Victoria, and the Catholic Education Office. Active informed parental consent was required at each data collection point. Parents and the older children completed self-administered questionnaires and children wore accelerometers for one week at each time-point.

### Measures

#### Active Transport

At each time-point, parents of the younger cohort reported how often per week their children walked/cycled to neighborhood destinations including school, sports facilities, shops and friends' homes [16,17]. The older cohort self-reported these frequencies. Frequency values (in parentheses) were assigned to each response category: (0) "not within walking/riding distance"; (0) "never/rarely"; (0.5) "less than once per week"; (1.5) "one to two times per week"; (3.5) "three to four times per week"; (5.5) "five to six times per week"; and (7) "daily". The number of active trips per week was computed, and moderate to high test-retest reliability was demonstrated over a one week period among 53 parents (ICC = 0.86) and 66 adolescents aged 13-15 years (ICC = 0.68).

#### Physical Activity

Physical activity was objectively monitored for eight consecutive days at each time point using a hip-mounted uni-axial accelerometer (Actigraph model 7164, Fort Walton Beach, Florida, USA). The epoch length was 60 seconds. Children were instructed to wear the accelerometer during all waking hours except during water-based activities (e.g. swimming, showering). Accelerometers are a valid objective monitor for assessing free-living physical activity in paediatric populations [18].

#### Data Management and Reduction

Data were downloaded according to manufacturer guidelines. The first day's recorded data were discarded. Data were processed using a customized Excel macro. Non-wear was defined as sustained bouts of 20 minutes of zero counts, and the total duration of these periods represented the duration for which the monitor had not been worn [19]. For inclusion in the analyses, each participant was required to have recorded counts for a minimum of 8 hours a day on at least 4 days (including a minimum of 1 weekend day) at each time point.

Age-specific cut-points [20] determined time spent in moderate physical activity (MPA; 4-5.99 METS; [21]) and vigorous physical activity (VPA; >6.0 METS). MPA and VPA were summed to obtain moderate-to-vigorous physical activity (MVPA). Time spent (minutes) engaged in MVPA per valid day by each participant was computed, as well as the mean time spent engaged in MVPA on week days and weekend days.

### Data Analyses

Analyses were conducted using SPSS v17 and Stata/SE v10 for participants with accelerometer data at all three time-points. Descriptive analyses were performed to examine the mean number of active trips and mean time spent in MVPA for boys and girls within each cohort at each time-point.

Tracking of active transport and of MVPA was examined over five years among boys and girls in each cohort using General Estimating Equations (GEE). Tracking of physical activity is considered 'low'; 'moderate' or 'high' if correlations between repeated measures have values less than 0.30, from 0.30 to 0.6 or above 0.6, respectively [8]. Stability coefficients were computed for each outcome variable using the methods of Twisk [22]. First, a first-order autoregressive model was utilized to predict the value of each variable at time t from the corresponding value at time t-1:

$$Y_{it}=\beta_{0}+\beta_{1}Y_{i\left(\text{t}-1\right)}+\varepsilon_{i\text{t}}$$

where *Y_it _*is the outcome variable value for subject i at time t, β_0 _is the intercept, β_1 _is the autoregression (tracking) coefficient, Y_i(t-1) _is the outcome variable value for subject i at time t - 1 and ε_it _is the error for subject i at time t. It was assumed all outcome variables had a Poisson distribution and their correlation patterns were unstructured. Second, tracking coefficients were standardized by applying the formula:

$$\beta_{s}=\beta \left(\frac{\text{sd}\left({\text{Y}}_{\text{t}-1}\right)}{\text{sd}\left({\text{Y}}_{\text{t}}\right)}\right)$$

where β_s _is the standardized tracking coefficient, β is the non-standardized tracking coefficient, sd(Y_t-1_) and sd(Y_t_) are the standard deviations of the outcome variables at times t-1 and t, respectively [[22], p. 229].

In addition, linear regression analyses were performed to examine how active transport was associated with MVPA at each time-point. Analyses were stratified by sex and by cohort.

## Results

Almost half (47%) of the baseline sample were boys. In most cases (82%) the parent survey was completed by the mother, and over one third (35%) of all mothers were tertiary educated. There were no significant differences between participants and non-participants at T2 according to maternal employment status, sex of the child/adolescent, or the number of times the child/adolescent walked to school per week. However, participants' mothers were more likely to be tertiary educated than not to have completed secondary school (OR = 1.99, 95% CI = 1.48 to 2.67). Furthermore, compared with non-participants at T2, participants, on average, engaged in 40 minutes more MVPA on week days (95% CI = 32 to 48) and 41 minutes more on weekend days (95% CI = 31 to 50) at baseline. When those who participated at T2 but not at T3 were compared with participants at T3, there were no significant differences in maternal education, maternal employment status, sex of the child/adolescent or the number of times the child/adolescent walked to school per week. In addition, there were no significant differences in mean MVPA recorded on week days at T2 by T3 participants compared with non-participants, but on average T3 participants engaged in 20 minutes more (95% CI = 1 to 38) MVPA on weekend days.

The mean number of active trips and mean duration of MVPA at each time-point for boys and girls in each cohort are presented in Table 1. With the exception of younger girls, on average, participants made more active trips at T3 than T1. However, there were declines in overall duration of MVPA on average on all days, on week days, and on weekends between T1 and T3.

**Table 1:** Mean (s.d.) number of active trips and minutes of moderate-to-vigorous physical activity at each time-point.

	Younger cohort		Older cohort	
	**Boys** **(n = 71)**	**Girls** **(n = 63)**	**Boys** **(n = 82)**	**Girls** **(n = 119)**

**Active trips (n)**				
T1	5.9 (4.4)	6.5 (6.0)	10.3 (6.4)	8.2 (6.9)
T2	6.2 (5.1)	5.2 (4.6)	12.8 (8.1)	10.2 (9.1)
T3	8.8 (7.1)	6.0 (5.3)	11.8 (7.2)	10.3 (8.3)
				
**MVPA daily (min)**				
T1	154.2 (34.4)	131.8 (30.4)	86.8 (42.8)	66.4 (33.0)
T2	117.5 (31.6)	88.6 (23.7)	59.1 (40.1)	40.9 (25.9)
T3	85.2 (29.4)	57.0 (20.4)	40.3 (17.8)	24.4 (14.8)
				
**MVPA on week days (min)**				
T1	156.6 (33.2)	135.1 (31.8)	89.6 (36.4)	71.0 (34.9)
T2	120.2 (30.0)	89.5 (24.0)	65.3 (46.2)	45.4 (28.7)
T3	89.7 (31.2)	60.0 (20.8)	46.0 (24.1)	27.0 (16.1)
				
**MVPA weekend days (min)**				
T1	147.9 (51.6)	125.3 (41.7)	70.1 (45.9)	51.4 (30.2)
T2	110.5 (51.2)	85.2 (39.0)	45.0 (42.8)	28.2 (28.6)
T3	72.8 (38.0)	48.5 (30.9)	25.4 (27.2)	15.5 (17.0)

Standardized tracking coefficients for active transport and MVPA are presented in Table 2. Active transport tracked moderately over five years among younger boys and girls, but did not track significantly among the older cohort. MVPA tracked moderately for boys and girls in both cohorts in each of the periods examined.

**Table 2:** Standardized tracking coefficients for active transport and moderate-to-vigorous physical activity stratified by cohort and sex

	Tracking (β) coefficients			
	**Younger cohort**		**Older cohort**	

	**Boys** **(n = 71)**	**Girls** **(n = 63)**	**Boys** **(n = 82)**	**Girls** **(n = 119)**

Active trips	0.36***	0.51***	0.06	0.10
MVPA daily	0.55***	0.49***	0.45***	0.36***
MVPA week days	0.53***	0.44***	0.49***	0.33***
MVPA - weekend days	0.47***	0.45***	0.38***	0.36***

*** p < 0.001 - p values refer to non-standardized coefficients

Cross-sectional associations between active transport and MVPA at each time-point are presented in Table 3. There were no significant associations between active transport and MVPA among younger boys and girls. At both T1 and T2, active transport was positively associated only with older boys' MVPA on week days and accounted for 6.0% and 4.9%, respectively, of the variance in MVPA. At T3, however, active transport was associated with (and accounted for 7.3% of the variance in) older boys' daily MVPA and with their MVPA on week days (accounting for 4.8% of the variance), as well as with older girls' daily MVPA, their MVPA on week days and on weekends. Active transport accounted for 5.7% of the variance in older girls' daily MVPA, 4.0% of the variance in their MVPA on week days, and 7.0% of the variance in their MVPA on weekends.

**Table 3:** Cross-sectional associations between number of active trips and MVPA at three time-points

	Regression coefficient (95% CI)			
	**Younger Boys**	**Younger Girls**	**Adolescent Boys**	**Adolescent Girls**

**T1**	**Age 5-6 years**	**Age 5-6 years**	**Age 10-12 years**	**Age 10-12 years**
MVPA daily	-1.46 (-3.31, 0.39)	0.21 (-1.08, 1.51)	1.06 (0.40, 2.52)	-0.09 (-0.96, 0.79)
MVPA week days	-1.15 (-2.95, 0.64)	0.43 (-0.91, 1.78)	1.37 (0.15, 2.59)*	0.07 (-0.86, 0.99)
MVPA weekends	-1.84 (-4.66, 0.97)	0.24 (-1.59, 2.06)	0.33 (-1.32, 1.98)	-0.48 (-1.04, 0.57)
				
**T2**	**Age 8-9 years**	**Age 8-9 years**	**Age 13-15 years**	**Age 13-15 years**
MVPA daily	0.94 (-0.51, 2.40)	-0.06 (-1.36, 1.25)	1.07 (-0.01, 2.15)	0.34 (-0.18, 0.86)
MVPA week days	0.78 (-0.61, 2.16)	0.10 (-1.23, 1.42)	1.27 (0.03, 2.51)*	0.55 (-0.02, 1.12)
MVPA weekends	1.60 (-0.78, 3.99)	-0.41 (-2.63, 1.81)	0.80 (-0.38, 1.98)	-0.11 (-0.70, 0.49)
				
**T3**	**Age 10-11 years**	**Age 10-11 years**	**Age 15-17 years**	**Age 15-17 years**
MVPA daily	0.59 (-0.39, 1.57)	0.69 (-0.26, 1.63)	0.67 (0.14, 1.20)*	0.44 (0.11, 0.77)*
MVPA week days	0.50 (-0.54, 1.54)	0.69 (-0.27, 1.65)	0.74 (0.01, 1.47)*	0.40 (0.04, 0.76)*
MVPA weekends	0.87 (-0.40, 2.15)	0.27 (-1.25, 1.79)	0.68 (-0.24, 1.60)	0.54 (0.16, 0.93)**

* p < 0.05; ** p < 0.01; ***, p < 0.001

## Discussion

This study is among the first to examine how active transport is associated with physical activity among children and adolescents over time and is particularly novel in examining tracking of active transport across childhood and adolescence. Among the strengths of this study is the inclusion of data (including objectively-measured physical activity) from two distinct age-groups, and the collection of data at three time-points over five years. While initial increases in active transport may not appear to reconcile decreases in physical activity levels, it is important to note (a limitation of the study) that only frequency of trips was examined in this study and not the length and duration of each walking/cycling trip. Two further limitations should be acknowledged. Firstly, as a range of accelerometer cut-points and MET values have been published in the literature to define MPA and VPA in youth, our findings are applicable only to the cut-points and the definition of MPA (>4 METs) used in this study. Secondly, hip-worn accelerometers may not accurately measure (and under-estimate) non-ambulatory activities such as cycling [23].

Active transport was shown to track moderately among the children but not among the adolescents. By contrast, physical activity tracked moderately over five years for all participant groups. One explanation of the lack of tracking of active transport among adolescents is that they made the transition from primary to secondary school between T1 to T2, whereas the younger cohort remained in primary school for the duration of the study. The transition from primary to secondary school often involves a change in physical location of schooling as well as variation in peer groups [24], both of which may influence active transport behaviours. At this time children are often granted greater independent mobility by their parents [1,6,24]. The increases in active transport among adolescents between T1 and T2 may be indicative of this, but the lack of tracking highlights that these behaviours are highly variable and amenable to change in response to external factors as participants did not maintain their relative rank. Active transport behaviours among children attending primary school appear quite stable, suggesting that it is important to develop appropriate strategies to promote and support these behaviours at the beginning of primary school.

While physical activity tracked moderately over time, our findings demonstrated that its association with active transport changed over time. Active transport became more strongly associated with MVPA during adolescence, possibly due to increased independent mobility. Active transport was associated more with boys' MVPA (than with girls' MVPA) at an earlier age, possibly because boys are granted independent mobility at an earlier age than are girls [1,25]. However, later in adolescence, there were more associations between active transport and MVPA across the whole week among girls. Therefore, active transport may be an important source of habitual physical activity for adolescent girls, among whom low and declining physical activity levels have been reported in developed countries [26-29], and the need to support this behaviour is paramount.

This research emphasizes the requirement for local government, urban planners and policy-makers to promote active transport through the design of the built environment [30,31]. From an urban planning perspective, in order to encourage active transport it is essential that appropriate walkable destinations such as shops, school, medical centres and recreational facilities are located in residential areas [32]. To further support walking and cycling, physical infrastructure such as bike paths and/or lanes, walking tracks and pedestrian crossings are required, as well as traffic-calming measures on residential streets to create pedestrian- and child-friendly environments [17,33].

## Conclusions

While active transport should be encouraged among all school-aged children, it is especially important to promote continuing this behaviour, during adolescence when overall physical activity levels have been shown to decrease, particularly among girls. There is also a need to promote other supplementary forms of physical activity in order to increase adolescents' overall physical levels. Tailored interventions may be required to ensure that adolescents continue to walk/cycle for transport once they are old enough (e.g. 18 years of age, in the State of Victoria, Australia) to obtain their drivers' licence. As well as promoting the health benefits of walking and cycling, interventions could emphasize the low cost of these transport modes compared with driving, and how beneficial sustainable transport is by reducing carbon emissions, noise pollution and dependence on fossil fuels [3] and promoting social interaction.

## Competing interests

The authors declare that they have no competing interests.

## Authors' contributions

AC was responsible for the overall conception and design of this manuscript, statistical analysis and interpretation of data. AT and JS were responsible for data acquisition, and contributed along with KH and DC to drafting and critical revision of the manuscript. NR processed the accelerometer data and was involved in drafting the 'Methods' section and in critical revision. All authors read and approved the final manuscript.
